# Supplementary material for: HIVprotI: an integrated web based platform for prediction and design of HIV proteins inhibitors
Source: J Cheminform. 2018 Mar 9;10:12. doi: 10.1186/s13321-018-0266-y (PMC5845081; doi:10.1186/s13321-018-0266-y)
Supplement: Supplementary file 2 — Additional file 2. Source code of HIVProtI web server. [file 13321_2018_266_MOESM2_ESM.zip › HIVprotI_Source-code/hivprotI/weka-3-6-6/doc/weka/associations/FPGrowth.AssociationRule.html]

FPGrowth.AssociationRule


---


|  |  |  |
| --- | --- | --- |
| **Overview** \| **Package** \| **Class** \| **Tree** \| **Deprecated** \| **Index** \| **Help** \| **Weka's home** | |  |
| **PREV CLASS**   **NEXT CLASS** | **FRAMES**    **NO FRAMES**     **All Classes** |  |
| SUMMARY: NESTED \| FIELD \| CONSTR \| METHOD | DETAIL: FIELD \| CONSTR \| METHOD |  |


---


## weka.associations Class FPGrowth.AssociationRule

```
java.lang.Object
  weka.associations.FPGrowth.AssociationRule
```

**All Implemented Interfaces:**: java.io.Serializable, java.lang.Comparable<FPGrowth.AssociationRule>

**Enclosing class:**: FPGrowth

---

``` public static class FPGrowth.AssociationRule extends java.lang.Object implements java.io.Serializable, java.lang.Comparable<FPGrowth.AssociationRule> ```

**Author:**
:   mhall

**See Also:**: Serialized Form

---

| **Nested Class Summary** | |
| --- | --- |
| `static class` | `FPGrowth.AssociationRule.METRIC_TYPE`             Enum for holding different metric types |


| **Field Summary** | |
| --- | --- |
| `static Tag[]` | `TAGS_SELECTION`             Tags for display in the GUI |


**Constructor Summary**

`FPGrowth.AssociationRule(java.util.Collection<FPGrowth.BinaryItem> premise,
java.util.Collection<FPGrowth.BinaryItem> consequence,
FPGrowth.AssociationRule.METRIC_TYPE metric,
int premiseSupport,
int consequenceSupport,
int totalSupport,
int totalTransactions)`
  
          Construct a new association rule.


| **Method Summary** | |
| --- | --- |
| `int` | `compareTo(FPGrowth.AssociationRule other)`             Compare this rule to the supplied rule. |
| `boolean` | `containsItems(java.util.ArrayList<Attribute> items, boolean useOr)` |
| `boolean` | `equals(java.lang.Object other)`             Return true if this rule is equal to the supplied one. |
| `static java.util.List<FPGrowth.AssociationRule>` | `generateRulesBruteForce(weka.associations.FPGrowth.FrequentItemSets largeItemSets, FPGrowth.AssociationRule.METRIC_TYPE metricToUse, double metricThreshold, int upperBoundMinSuppAsInstances, int lowerBoundMinSuppAsInstances, int totalTransactions)`             Generate all association rules, from the supplied frequet item sets, that meet a given minimum metric threshold. |
| `java.util.Collection<FPGrowth.BinaryItem>` | `getConsequence()`             Get the consequence of this rule. |
| `int` | `getConsequenceSupport()`             Get the support for the consequence. |
| `FPGrowth.AssociationRule.METRIC_TYPE` | `getMetricType()`             Get the metric type of this rule (e.g. |
| `double` | `getMetricValue()`             Get the value of the metric for this rule. |
| `java.util.Collection<FPGrowth.BinaryItem>` | `getPremise()`             Get the premise of this rule. |
| `int` | `getPremiseSupport()`             Get the support for the premise. |
| `int` | `getTotalSupport()`             Get the total support for this rule (premise + consequence). |
| `int` | `getTotalTransactions()`             Get the total number of transactions in the data. |
| `static java.util.List<FPGrowth.AssociationRule>` | `pruneRules(java.util.List<FPGrowth.AssociationRule> rulesToPrune, java.util.ArrayList<Attribute> itemsToConsider, boolean useOr)` |
| `java.lang.String` | `toString()`             Get a textual description of this rule. |
| `java.lang.String` | `toXML()` |

**Methods inherited from class java.lang.Object**

`getClass, hashCode, notify, notifyAll, wait, wait, wait`

**Field Detail**

### TAGS\_SELECTION

```
public static final Tag[] TAGS_SELECTION
```

:   Tags for display in the GUI


**Constructor Detail**

### FPGrowth.AssociationRule

```
public FPGrowth.AssociationRule(java.util.Collection<FPGrowth.BinaryItem> premise,
                                java.util.Collection<FPGrowth.BinaryItem> consequence,
                                FPGrowth.AssociationRule.METRIC_TYPE metric,
                                int premiseSupport,
                                int consequenceSupport,
                                int totalSupport,
                                int totalTransactions)
```

:   Construct a new association rule.

    **Parameters:**: `premise` - the premise of the rule: `consequence` - the consequence of the rule: `metric` - the metric for the rule: `premiseSupport` - the support of the premise: `consequenceSupport` - the support of the consequence: `totalSupport` - the total support of the rule: `totalTransactions` - the number of transactions in the data


**Method Detail**

### getPremise

```
public java.util.Collection<FPGrowth.BinaryItem> getPremise()
```

:   Get the premise of this rule.

    :   **Returns:**: the premise of this rule.

---


### getConsequence

```
public java.util.Collection<FPGrowth.BinaryItem> getConsequence()
```

:   Get the consequence of this rule.

    :   **Returns:**: the consequence of this rule.

---


### getMetricType

```
public FPGrowth.AssociationRule.METRIC_TYPE getMetricType()
```

:   Get the metric type of this rule (e.g. confidence).

    :   **Returns:**: the metric type of this rule.

---


### getMetricValue

```
public double getMetricValue()
```

:   Get the value of the metric for this rule.

    :   **Returns:**: the value of the metric for this rule.

---


### getPremiseSupport

```
public int getPremiseSupport()
```

:   Get the support for the premise.

    :   **Returns:**: the support for the premise.

---


### getConsequenceSupport

```
public int getConsequenceSupport()
```

:   Get the support for the consequence.

    :   **Returns:**: the support for the consequence.

---


### getTotalSupport

```
public int getTotalSupport()
```

:   Get the total support for this rule (premise + consequence).

    :   **Returns:**: the total support for this rule.

---


### getTotalTransactions

```
public int getTotalTransactions()
```

:   Get the total number of transactions in the data.

    :   **Returns:**: the total number of transactions in the data.

---


### compareTo

```
public int compareTo(FPGrowth.AssociationRule other)
```

:   Compare this rule to the supplied rule.

    :   **Specified by:**: `compareTo` in interface `java.lang.Comparable<FPGrowth.AssociationRule>`
    :   **Parameters:**: `other` - the rule to compare to. **Returns:**: the result of the comparison.

---


### equals

```
public boolean equals(java.lang.Object other)
```

:   Return true if this rule is equal to the supplied one.

    :   **Overrides:**: `equals` in class `java.lang.Object`
    :   **Returns:**: true if this rule is the same as the supplied rule.

---


### containsItems

```
public boolean containsItems(java.util.ArrayList<Attribute> items,
                             boolean useOr)
```

---


### toString

```
public java.lang.String toString()
```

:   Get a textual description of this rule.

    :   **Overrides:**: `toString` in class `java.lang.Object`
    :   **Returns:**: a textual description of this rule.

---


### toXML

```
public java.lang.String toXML()
```

---


### generateRulesBruteForce

```
public static java.util.List<FPGrowth.AssociationRule> generateRulesBruteForce(weka.associations.FPGrowth.FrequentItemSets largeItemSets,
                                                                               FPGrowth.AssociationRule.METRIC_TYPE metricToUse,
                                                                               double metricThreshold,
                                                                               int upperBoundMinSuppAsInstances,
                                                                               int lowerBoundMinSuppAsInstances,
                                                                               int totalTransactions)
```

:   Generate all association rules, from the supplied frequet item sets,
    that meet a given minimum metric threshold. Uses a brute force approach.

    :   **Parameters:**: `largeItemSets` - the set of frequent item sets: `metricToUse` - the metric to use: `metricThreshold` - the threshold value that a rule must meet: `upperBoundMinSuppAsInstances` - the upper bound on the support in order to accept the rule: `lowerBoundMinSuppAsInstances` - the lower bound on the support in order to accept the rule: `totalTransactions` - the total number of transactions in the data **Returns:**: a list of association rules

---


### pruneRules

```
public static java.util.List<FPGrowth.AssociationRule> pruneRules(java.util.List<FPGrowth.AssociationRule> rulesToPrune,
                                                                  java.util.ArrayList<Attribute> itemsToConsider,
                                                                  boolean useOr)
```


---


|  |  |  |
| --- | --- | --- |
| **Overview** \| **Package** \| **Class** \| **Tree** \| **Deprecated** \| **Index** \| **Help** \| **Weka's home** | |  |
| **PREV CLASS**   **NEXT CLASS** | **FRAMES**    **NO FRAMES**     **All Classes** |  |
| SUMMARY: NESTED \| FIELD \| CONSTR \| METHOD | DETAIL: FIELD \| CONSTR \| METHOD |  |


---
